# Association of a Marker of *N*-Acetylglucosamine With Progressive Multiple Sclerosis and Neurodegeneration

**DOI:** 10.1001/jamaneurol.2021.1116

**Published:** 2021-05-10

**Authors:** Alexander U. Brandt, Michael Sy, Judith Bellmann-Strobl, Barbara L. Newton, Judy Pawling, Hanna G. Zimmermann, Zhaoxia Yu, Claudia Chien, Jan Dörr, Jens Th. Wuerfel, James W. Dennis, Friedemann Paul, Michael Demetriou

**Affiliations:** 1 Experimental and Clinical Research Center, Charité–Universitätsmedizin Berlin, corporate member of Freie Universität Berlin and Humboldt-Universität zu Berlin, and Max Delbrück Center for Molecular Medicine, Berlin, Germany; 2 Department of Neurology, University of California, Irvine, Irvine; 3 NeuroCure Clinical Research Center, Charité–Universitätsmedizin Berlin, corporate member of Freie Universität Berlin and Humboldt-Universität zu Berlin, Berlin, Germany; 4 Samuel Lunenfeld Research Institute, Mount Sinai Hospital, Toronto, Toronto, Ontario, Canada; 5 Department of Statistics, University of California, Irvine, Irvine; 6 Medical Image Analysis Center, Department of Biomedical Engineering, University Basel, Basel, Switzerland; 7 Department of Molecular Genetics, University of Toronto, Toronto, Ontario, Canada; 8 Department of Microbiology and Molecular Genetics, University of California, Irvine, Irvine

## Abstract

**Importance:**

*N*-glycan branching modulates cell surface receptor availability, and its deficiency in mice promotes inflammatory demyelination, reduced myelination, and neurodegeneration. *N*-acetylglucosamine (GlcNAc) is a rate-limiting substrate for *N*-glycan branching, but, to our knowledge, endogenous serum levels in patients with multiple sclerosis (MS) are unknown.

**Objective:**

To investigate a marker of endogenous serum GlcNAc levels in patients with MS.

**Design, Setting, and Participants:**

A cross-sectional discovery study and cross-sectional confirmatory study were conducted at 2 academic MS centers in the US and Germany. The discovery study recruited 54 patients with MS from an outpatient clinic as well as 66 healthy controls between April 20, 2010, and June 21, 2013. The confirmatory study recruited 180 patients with MS from screening visits at an academic MS study center between April 9, 2007, and February 29, 2016. Serum samples were analyzed from December 2, 2013, to March 2, 2015. Statistical analysis was performed from February 23, 2020, to March 18, 2021.

**Main Outcomes and Measures:**

Serum levels of GlcNAc plus its stereoisomers, termed *N*-acetylhexosamine (HexNAc), were assessed using targeted tandem mass spectroscopy. Secondary outcomes (confirmatory study) comprised imaging and clinical disease markers.

**Results:**

The discovery cohort included 66 healthy controls (38 women; mean [SD] age, 42 [20] years), 33 patients with relapsing-remitting MS (RRMS; 25 women; mean [SD] age, 50 [11] years), and 21 patients with progressive MS (PMS; 14 women; mean [SD] age, 55 [7] years). The confirmatory cohort included 125 patients with RRMS (83 women; mean [SD] age, 40 [9] years) and 55 patients with PMS (22 women; mean [SD] age, 49 [80] years). In the discovery cohort, the mean (SD) serum level of GlcNAc plus its stereoisomers (HexNAc) was 710 (174) nM in healthy controls and marginally reduced in patients with RRMS (mean [SD] level, 682 [173] nM; *P* = .04), whereas patients with PMS displayed markedly reduced levels compared with healthy controls (mean [SD] level, 548 [101] nM; *P* = 9.55 × 10^−9^) and patients with RRMS (*P* = 1.83 × 10^−4^). The difference between patients with RRMS (mean [SD] level, 709 [193] nM) and those with PMS (mean [SD] level, 405 [161] nM; *P* = 7.6 × 10^−18^) was confirmed in the independent confirmatory cohort. Lower HexNAc serum levels correlated with worse expanded disability status scale scores (ρ = –0.485; *P* = 4.73 × 10^−12^), lower thalamic volume (*t* = 1.7; *P* = .04), and thinner retinal nerve fiber layer (*B* = 0.012 [SE = 7.5 × 10^−11^]; *P* = .008). Low baseline serum HexNAc levels correlated with a greater percentage of brain volume loss at 18 months (*t* = 1.8; *P* = .04).

**Conclusions and Relevance:**

This study suggests that deficiency of GlcNAc plus its stereoisomers (HexNAc) may be a biomarker for PMS. Previous preclinical, human genetic, and ex vivo human mechanistic studies revealed that *N*-glycan branching and/or GlcNAc may reduce proinflammatory responses, promote myelin repair, and decrease neurodegeneration. Combined, the data suggest that GlcNAc deficiency may be associated with progressive disease and neurodegeneration in patients with MS.

## Key Points

**Question:**

Is the serum concentration of *N*-acetylglucosamine (GlcNAc) altered in patients with multiple sclerosis?

**Findings:**

This cross-sectional study found that patients with a progressive multiple sclerosis subtype and more severe disease have reduced serum levels of a marker of GlcNAc. In addition, GlcNAc is a rate-limiting substrate for *N*-glycan branching, which has been shown to regulate immunoactivity and myelination.

**Meaning:**

This study suggests that GlcNAc and *N*-glycan branching are associated with multiple sclerosis in general and progressive multiple sclerosis in particular.

## Teaser

This cross-sectional study investigates a marker of endogenous serum *N*-acetylglucosamine levels in patients with multiple sclerosis (MS).

## Introduction

Multiple sclerosis (MS) is characterized by recurrent episodes of neurologic dysfunction resulting from acute inflammatory demyelination.^1^ Progressive MS (PMS) is distinguished by continuous inflammation, failure to remyelinate, and progressive neurodegeneration, causing accrual of irreversible neurologic disability. After approximately 20 years, relapsing-remitting MS (RRMS) converts to secondary-progressive MS (SPMS) for many patients, while approximately 10% of patients have a primary progressive disease course from onset (PPMS).^2^ However, disease progression outside relapses is not limited to the progressive forms of the disease; instead, it is an inherent feature from the early stages and throughout all disease courses.^3^

**Figure 1.**
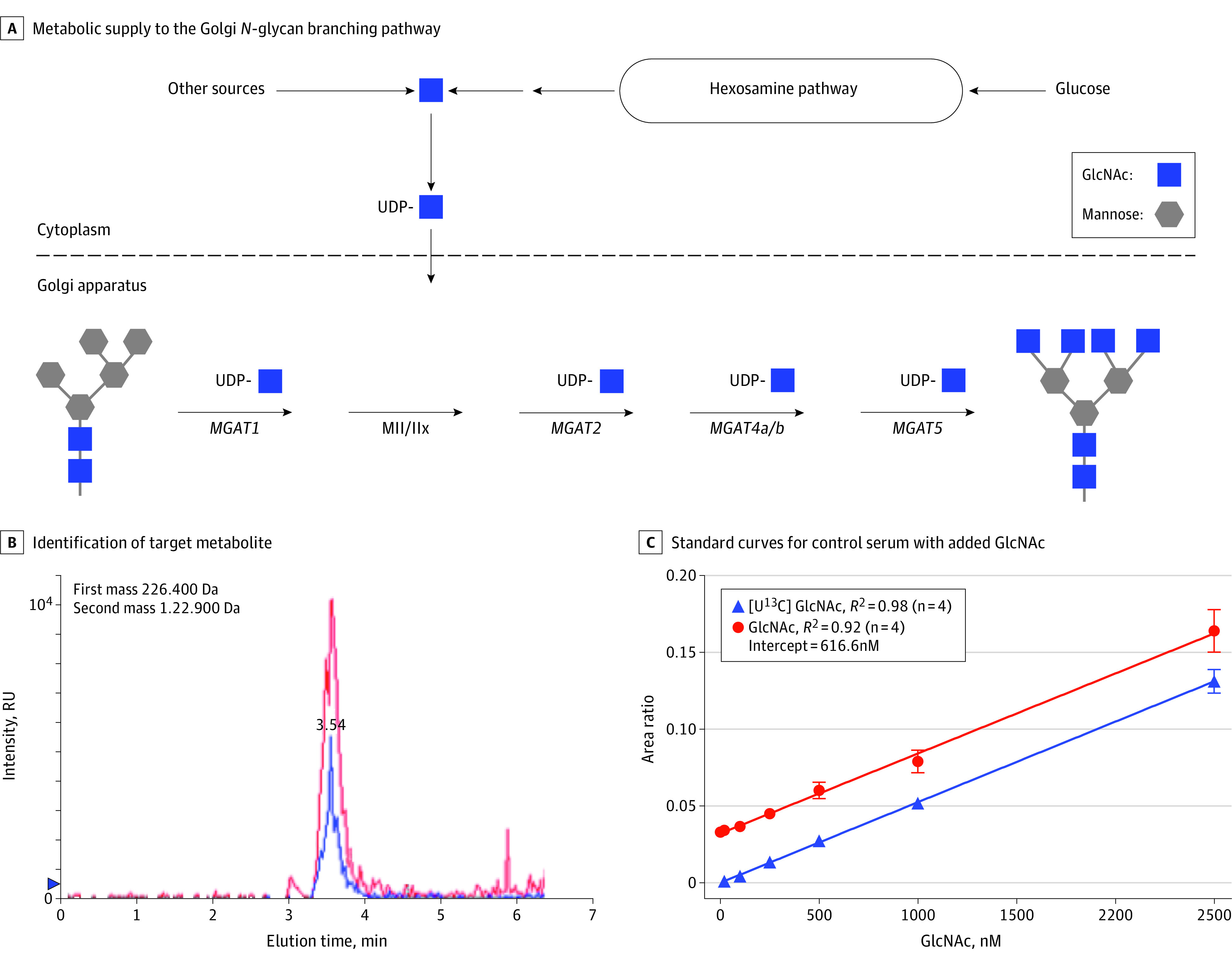
Analysis of Serum *N*-Acetylglucosamine (GlcNAc) by Liquid Chromatography–Tandem Mass Spectroscopy A, Schematic overview of metabolic supply to the Golgi *N*-glycan branching pathway. Uridine diphosphate (UDP)–GlcNAc, via de novo synthesis from glucose or salvage from GlcNAc, is the donor substrate used by the Mgat branching enzymes. B, The primary mass identifies the target metabolite, and the secondary or fragmented ion at time of liquid chromatography elution, as shown, is used to quantify *N*-acetylhexosamine (HexNAc). In the same run, endogenous serum HexNAc (red) and 500nM U^13^C-labeled GlcNAc standard (blue) are shown. The area under the peak for the fragmented ion is normalized to the internal standard, D^7^-glucose, added during sample preparation. C, Standard curves for control serum (39 individuals pooled) with either unlabeled GlcNAc or U^13^C-labeled GlcNAc at increasing concentrations added to the serum. The U^13^C-labeled GlcNAc shows measurement sensitivity to 20nM and confirms linearity to this concentration.

Previous studies have indicated the potential relevance of *N*-glycosylation in MS.^4,5,6,7,8,9,10,11,12,13,14^ Proteins are posttranslationally modified by the addition of complex sugars (glycans), thereby creating glycoproteins. Modification of cell surface receptors and transporters with branched *N*-glycans via *N*-glycosylation coordinates cell growth and differentiation by controlling glycoprotein clustering, signaling, and endocytosis.^4,5,6,7,8,9,10,11,12,13,14^ A critical metabolic precursor in *N*-glycosylation and branching is *N*-acetylglucosamine (GlcNAc), a common amino sugar that is part of the regular human diet.^6,7,11,15,16^ Extracellular GlcNAc enters cells through macropinocytosis and supplements the de novo hexosamine biosynthesis pathway to uridine diphosphate (UDP)–GlcNAc, the donor substrate for branching *N*-acetylglucosaminyltransferases, encoded by the *MGAT* gene family (Figure 1A).

Reduction in *N*-glycan branching in mice promotes T-cell–mediated and B-cell–mediated inflammatory demyelination,^4,13,15,16,17,18^ as well as independently blocking myelin repair via inhibition of remyelination from oligodendrocyte precursor cells.^19^ Human sequence variants and environmental factors associated with MS alter *N*-glycan branching, including interleukin 7 receptor α, interleukin 2 receptor α, *MGAT1* (OMIM 160995), *MGAT5* (OMIM 601774), and vitamin D_3_.^20,21,22,23^ In PL/J mice, branching deficiency induces a spontaneous and slowly progressive demyelinating disease that mimics PMS, characterized by axonal damage and neuronal death in otherwise normal-appearing white and gray matter along with inflammatory demyelination.^17^ Neuron-specific deletion of *Mgat1* leads to spontaneous neuron apoptosis and a severe neurologic clinical syndrome in adult mice.^24^ Oral GlcNAc supplementation in murine models of MS inhibits pro-autoimmune T-cell and B-cell responses, drives myelin repair, and ameliorates clinical disease by enhancing *N*-glycan branching via increased UDP-GlcNAc supply to Golgi branching enzymes.^6,11,15,16^ In mice, oral GlcNAc is taken up and found in serum at a rate comparable to that of glucose.^25^ In addition, humans with loss-of-function variants in *PGM3* (OMIM 172100), a gene required to generate UDP-GlcNAc de novo or from GlcNAc, display reduced *N*-glycan branching, severe central nervous system hypomyelination, and autoimmunity.^26,27^

Despite this converging evidence from environmental factors, genetic sequence variants, and animal models of MS, endogenous serum GlcNAc levels have not been established in humans or investigated in association with MS, to our knowledge. Here, we used a unique targeted liquid chromatography–tandem mass spectroscopy (LC-MS/MS) approach with ion pairing to assess serum levels of GlcNAc plus its stereoisomers (*N*-acetylhexosamine [HexNAc]) in healthy controls and patients with MS. Furthermore, we investigate whether serum levels of HexNAc correlate with a progressive disease course, severity, and neurodegeneration in MS.

## Methods

### Discovery Cohort

A total of 54 non-Latino White patients with MS and 66 healthy controls (HC) were recruited between April 20, 2010, and June 21, 2013, from the MS outpatient clinic at the Institute for Clinical and Translational Sciences at University of California, Irvine, as well as from the 90+ Study Cohort of people aged 90 years or older from Laguna Woods, California (eTable 1 in the Supplement), originally as part of either genetic and/or immune aging studies. Inclusion criteria for patients with MS in this analysis were RRMS according to the 2010 revised McDonald criteria^28^ and progressive disease course based on the 1996 criteria by Lublin et al.^29^ Exclusion criteria were types 1 and 2 diabetes, kidney disease (elevated creatinine level), use of an oral glucosamine or GlcNAc supplement, and relapse within the last 3 months. Disease course was derived from clinical information. Blood samples were obtained randomly in regard to food intake. Written informed consent was obtained from all participants as part of a protocol reviewed and approved by the University of California, Irvine institutional review board.

### Confirmatory Cohort

A total of 180 patients with MS were recruited from the neuroimmunology clinical trial unit at the NeuroCure Clinical Research Center, Charité–Universitätsmedizin Berlin in Berlin, Germany, between April 9, 2007, and February 29, 2016, from screening or baseline visits from 2 interventional trials (eTable 2 in the Supplement). Inclusion criteria were MS based on the 2005 revised McDonald criteria,^30^ stable immunomodulatory therapy with glatiramer acetate (for RRMS), or no treatment (for PPMS and SPMS). Exclusion criteria were acute relapse and/or use of corticosteroids within 6 months prior to inclusion. Disease course was determined under strict adherence to the 1996 criteria by Lublin et al.^29^ Blood samples were obtained while participants were fasting. All study participants gave written informed consent on protocols reviewed and approved by the local Berlin ethics committee.

### Clinical Scoring

Clinical examination was performed according to the Expanded Disability Status Scale (EDSS) in Kurtzke.^31^ In addition, disability was assessed using a MS functional composite (MSFC), comprising the Timed 25-Foot Walk Test, the 9-Hole Peg Test, and the 3-second Paced Auditory Serial Additions Test^32^; MSFC *z* scores were calculated according to the MSFC Administration and Scoring Manual.^33^ Multiple Sclerosis Severity Scores were calculated from disease duration and the EDSS.^34^

### Magnetic Resonance Imaging

Magnetic resonance imaging (MRI) was performed at 1.5 T using 3-dimensional T1-weighted magnetization prepared rapid acquisition and multiple gradient echo sequences (MPRAGEs) and axial T2-weighted sequences. Images were either acquired on a Sonata MRI (Siemens Medical Systems) or on an Avanto MRI (Siemens Medical Systems) (eMethods in the Supplement).

Thalamic volume was determined as the summary volume from both hemispheres using FIRST (FSL, version 5.0; FMRIB Software Library)^35^ on MPRAGE scans and normalized using a brain-size normalization factor output from FSL SIENAX (FSL, version 5.0, FMRIB Software Library)^36^ for each brain. Brain volumes were determined using MPRAGE scans with the FSL, version 5.0 pipeline SIENAX (eMethods in the Supplement). SIENAX computes global brain volume (normalized brain volume) as well as normalized gray matter volume and normalized white matter volume estimates normalized with respect to the individual’s head size, accounting for interindividual variability. A 2-point percentage change in brain volume was estimated with SIENA, part of FSL,^36^ in patients for whom 18-month follow-up data were available. A pathologic percentage change in brain volume in patients was determined by an established cutoff of 0.52% annual loss (95% specificity).^37^

### Optical Coherence Tomography

Retinal nerve fiber layer thickness (RNFL) from both eyes was measured with a Stratus optical coherence tomography (OCT) device, software version 4.0 (Carl Zeiss Meditec) using the fast RNFL 3.4 protocol as previously described.^38^ Patients were examined without pupil dilation. Only images with acceptable quality were included in the analysis, defined as a visually even signal distribution, a reflectance signal strong enough to identify the RNFL layer borders, correct centration, and a signal strength of 7 or more of 10. Images with erroneous RNFL segmentation were excluded from analysis.

### Targeted LC-MS/MS

All serum samples were analyzed from December 2, 2013, to March 2, 2015, in a blinded fashion by the central mass spectroscopy laboratory in Toronto, Ontario, Canada. The operator performing the measurements (J.P.) was not involved in study design or informed about the scientific objectives. Results were then sent to the main investigators, who performed statistical analysis. Serum samples for metabolomics analysis were prepared as described previously (eMethods in the Supplement).^39^ Analysts were blinded in regard to sample origin (control or patient).

### Statistical Analysis

Statistical analysis was performed from February 23, 2020, to March 18, 2021, with R, version 3.5.3 (R Group for Statistical Computing). Sample sizes were based on convenience samples. Correlation between age or sex and serum HexNAc levels were analyzed with linear regression models for age and the Welch *t* test for sex in HC. Group differences between HC, patients with RRMS, and patients with PMS were analyzed using linear models with age as a covariate. To combine results of group comparisons in the discovery and confirmatory cohorts, we used the Fisher combined probability test, which combines *P* values using their logarithmic transformation. The association between treatment status and serum HexNAc level was assessed by use of the Kruskal-Wallis test. For boxplots, the solid middle line represents the median. The lower and upper hinges correspond to the 25th and 75th percentiles, respectively. The upper and lower whiskers extend from the hinge to the distant value no further than 1.5 × the interquartile range from the hinge. Receiver operating characteristic (ROC) curves were used to quantify the ability of serum HexNAc to discriminate between RRMS and PMS. Correlations between serum HexNAc level and clinical scores or imaging parameters were analyzed using linear regression models with HexNAc serum level as an independent parameter, except for EDSS and lesion measurements, which were analyzed using nonparametric Spearman ρ analyses (EDSS because of the measure’s ordinality and lesion measurements because of their nonnormal or skewed distributions [ie, as count variables]). Retinal nerve fiber layer correlations were analyzed with generalized estimating equation models accounting for intereye within-patient effects and using serum HexNAc level as an independent parameter. All correlation analyses based on parametric models were corrected for age and sex. Partial *R*^2^ was calculated to include only serum HexNAc-attributable variance in these models. Percentage change in brain volume between month 18 and baseline was compared using linear models with baseline normalized brain volume, age, and sex as covariates. Serum HexNAc concentrations before and during oral treatment with GlcNAc were compared using 1-sided paired Wilcoxon signed rank tests, comparing the mean of the weeks before treatment to the mean of the weeks receiving treatment. Data from the discovery and confirmatory cohorts were examined for nonnormal distributions by visual inspection of histograms and calculation of skewness and kurtosis. In case data were missing, data were not amended (sample size is indicated in each figure). Significance in all tests was established at *P* < .05. After initial 2-sided testing of HexNAc serum concentrations in HC and in patients with RRMS vs patients with PMS, all further tests were 1-sided, testing an association of serum HexNAc levels with worse disease outcomes.

## Results

### Detection of GlcNAc and Its Stereoisomers in HCs

Although GlcNAc is ingested daily by humans through normal dietary consumption, the availability of GlcNAc in human blood is poorly characterized, in part because levels are below detection in standard colorimetric assays. Therefore, to explore GlcNAc levels in human serum, we used LC-MS/MS with ion pairing.^39^ Mass detection via this method does not separate GlcNAc from its stereoisomers *N*-acetylgalactosamine (GalNAc) and *N*-acetylmannosamine (ManNAc); therefore, results are reported as HexNAc levels to reflect this. Detection of HexNAc by LC-MS/MS accurately tracks GlcNAc levels when added to human serum (Figure 1B and C).

In HCs from the discovery cohort (n = 66; 38 women and 28 men; mean [SD] age, 42 [20] years) (eTable 1 in the Supplement), HexNAc was readily detected, with a mean (SD) concentration of 710 (174) nM (range, 452nM-1374nM). The HexNAc concentration increased with age, with a mean increase of 4.8nM per year (*t* = 3.0; *P* < .001). There was no difference in serum concentration between male and female HCs (*t* = −0.7; *P* = .47).

### Detection of GlcNAc and Its Stereoisomers in Patients With PMS

**Figure 2.**
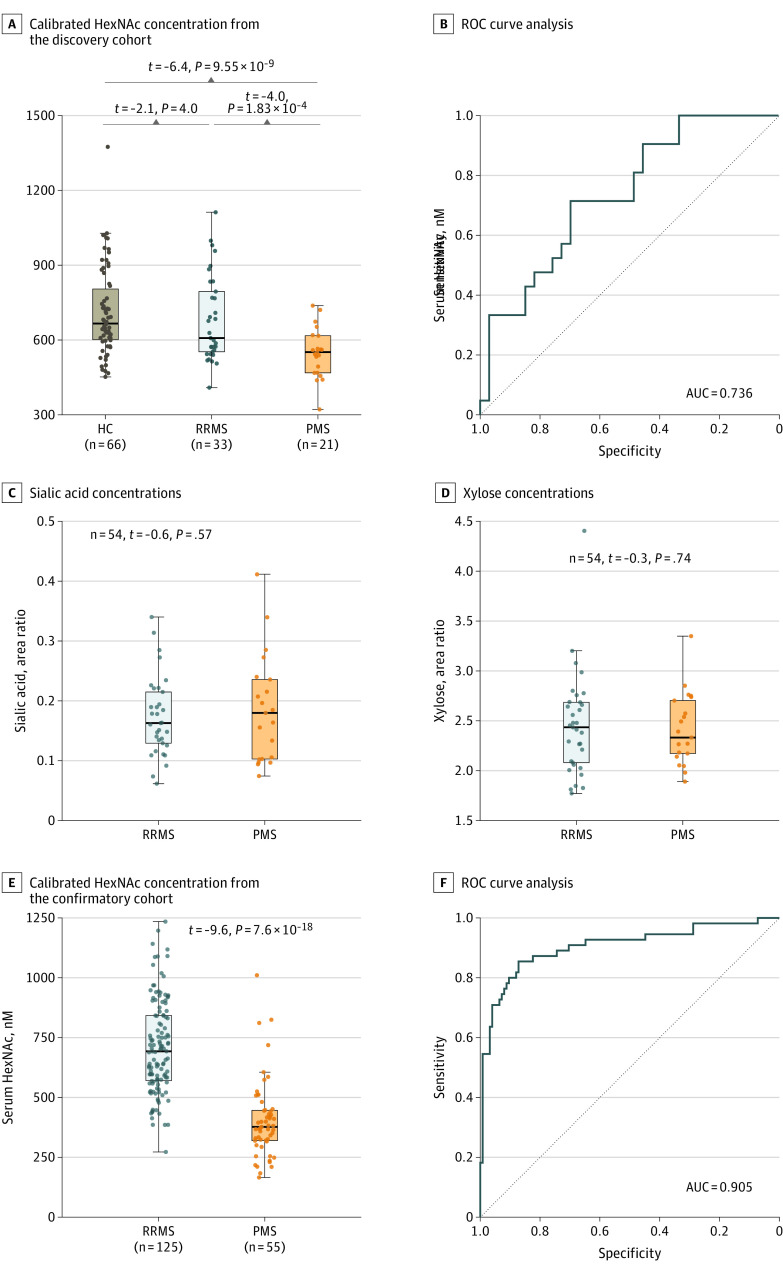
Reduction in Serum *N*-Acetylhexosamine (HexNAc) Level in Progressive Multiple Sclerosis A, Comparison of calibrated HexNAc concentration in serum samples from the discovery cohort. B, Receiver operating characteristic (ROC) curve analysis corresponding to panel A. C, Comparison of uncalibrated serum concentrations (area ratio [area of analyte/area of internal standard]) for control monosaccharide sialic acid in the discovery cohort. D, Comparison of uncalibrated serum concentrations for control monosaccharide xylose in the discovery cohort. E, Comparison of calibrated HexNAc concentration in serum samples from the confirmatory cohort. F, ROC curve analysis corresponding to panel E. The *t* values and *P* values are from linear regression models correcting for age. The area under the curve (AUC) is from ROC curve analysis. HC indicates healthy controls; PMS, progressive multiple sclerosis; and RRMS, relapsing-remitting multiple sclerosis.

To investigate a potential role for endogenous GlcNAc in both RRMS and PMS, we compared HC data with data on sera from 33 patients with RRMS (25 women and 8 men; mean [SD] age, 50 [11] years) and 21 patients with PMS (14 women and 7 men; mean [SD] age, 55 [7] years) (eTable 1 in the Supplement). With age as a covariate, patients with RRMS displayed slightly reduced HexNAc serum levels (mean [SD] level, 682 [173] nM) compared with HCs (*P* = .04), whereas patients with PMS had markedly reduced HexNAc serum levels (mean [SD] level, 548 [101] nM) compared with both HCs (*P* = 9.55 × 10^−9^) and patients with RRMS (*P* = 1.83 × 10^−4^) (Figure 2A). In a sensitivity analysis including only patients younger than 60 years, these associations were confirmed for patients with PMS (n = 17) vs HCs (n = 57) (*B* = –235.6 [SE = 43.9]; *P* = 9.7 × 10^−7^) or patients with RRMS (n = 27) (*B* = 182.4 [SE = 43.4]; *P* < .001), as well as patients with RRMS vs HCs (*B* = –76.8 [SE = 37.0]; *P* = .04). HexNAc levels were not associated with treatment status among patients with MS (n = 54; Kruskal-Wallis χ^2^ = 3.4; *P* = .34). In a ROC curve analysis, HexNAc levels discriminated between patients with RRMS and patients with PMS, with an area under the curve (AUC) of 0.736 (Figure 2B). In contrast to HexNAc, no differences in serum levels were observed between the groups for the monosaccharides sialic acid (mean [SD] level [AUC], 0.173 [0.066] in patients with RRMS vs 0.185 [0.089] in patients with PMS; *P* = .57) and xylose (mean [SD] level, 2.45 [0.51] in patients with RRMS vs 2.41 [0.36] in patients with PMS; *P* = .74), with the latter not involved in *N*-glycosylation (Figure 2C and D).

To confirm these findings, we analyzed an independent cohort of 180 patients with MS (125 RRMS; mean [SD] age, 40 [9] years; 83 women; and 55 PMS; mean [SD] age, 49 [8] years; 22 women) (eTable 2 in the Supplement). Based on our findings in the discovery cohort, a sample size of 50 would have been sufficient to confirm the observed difference in GlcNAc level between patients with RRMS and patients with PMS (95% power and α = 0.05, G × Power *t* test for detecting differences between 2 independent means). As in the initial cohort, HexNAc serum levels were greatly reduced in patients with PMS (mean [SD] level, 405 [161] nM; n = 55) vs those with RRMS (mean [SD] level, 709 [193] nM; n = 125; *P* = 7.6 × 10^−18^) (Figure 2E) and were readily discriminative between patients with RRMS and those with PMS (ROC AUC = 0.905; Figure 2F). HexNAc levels between patients with PPMS (mean [SD] level, 439 [219] nM; n = 23) and those with SPMS (mean [SD] level, 381 [99] nM; n = 32) were similar (*P* = .20). The serum HexNAc levels among patients with RRMS and those with PMS in the confirmatory cohort were similar to those in the initial cohort, despite the former being fasting and the latter being nonfasting. Combining *P* values from the initial and confirmatory cohort using the Fisher combined probability test results in *P* = 1.96 × 10^−20^ for PMS vs RRMS.

### Correlation of Serum GlcNAc and Its Stereoisomers With Disability and Imaging Markers of Neurodegeneration

**Figure 3.**
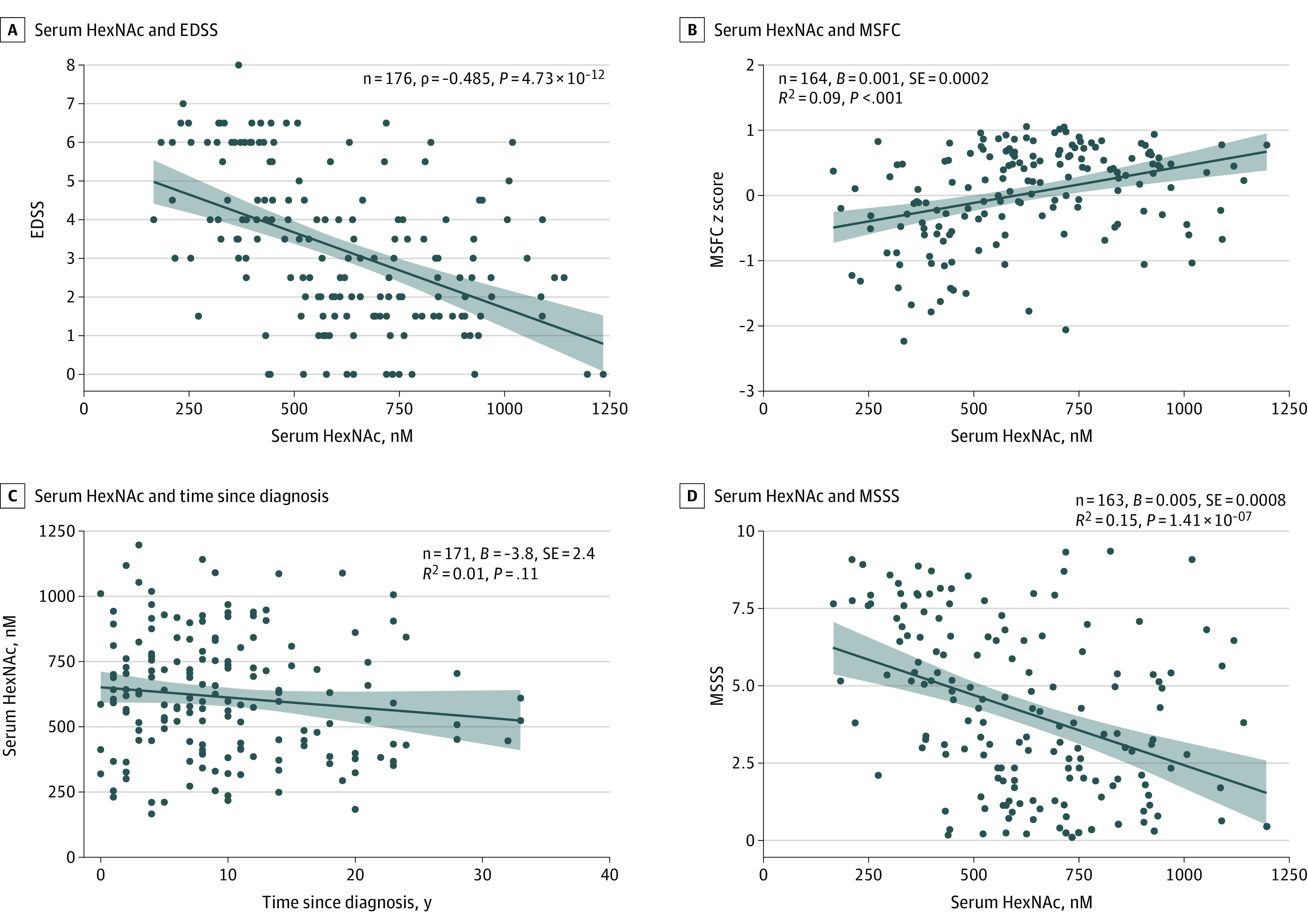
Correlation of Serum *N*-Acetylhexosamine (HexNAc) Level With Clinical Severity Markers A, Correlation between serum HexNAc level and Expanded Disability Status Scale (EDSS) score in the confirmatory cohort. B, Correlation between serum HexNAc level and multiple sclerosis functional composite (MSFC) *z* score in the confirmatory cohort. C, Correlation between serum HexNAc level and time since diagnosis. D, Correlation between serum HexNAc level and Multiple Sclerosis Severity Scale (MSSS) score. Coefficient *B*, SE, dark blue regression line, and light blue area 95% CI are from uncorrected linear regression models.

We further investigated the confirmatory cohort for the association of HexNAc levels with established clinical measures of disease severity and disability (namely, the EDSS and the MSFC scores). Lower HexNAc serum levels were associated with greater clinical disability displayed by higher EDSS scores (Figure 3A; *P* = 4.73 × 10^−12^) and lower MSFC scores (Figure 3B; *P* = 8.2 × 10^−5^). In linear models correcting for age and sex, serum HexNAc levels were associated with scores on the Timed 25-Foot Walk Test (*t* = −4; *P* = 5.8 × 10^−5^) and mean 9HPT scores (*t* = −2.6; *P* < .001) but not scores on the 3-second Paced Auditory Serial Additions Test (*t* = 0.973; *P* = .33). There was no correlation between lower HexNAc serum levels and time since diagnosis (Figure 3C; *P* = .114); however, the Multiple Sclerosis Severity Score, a disease severity parameter that combines EDSS and disease duration, was correlated inversely with serum HexNAc levels (Figure 3D, *P* = 1.41 × 10^−7^).

**Figure 4.**
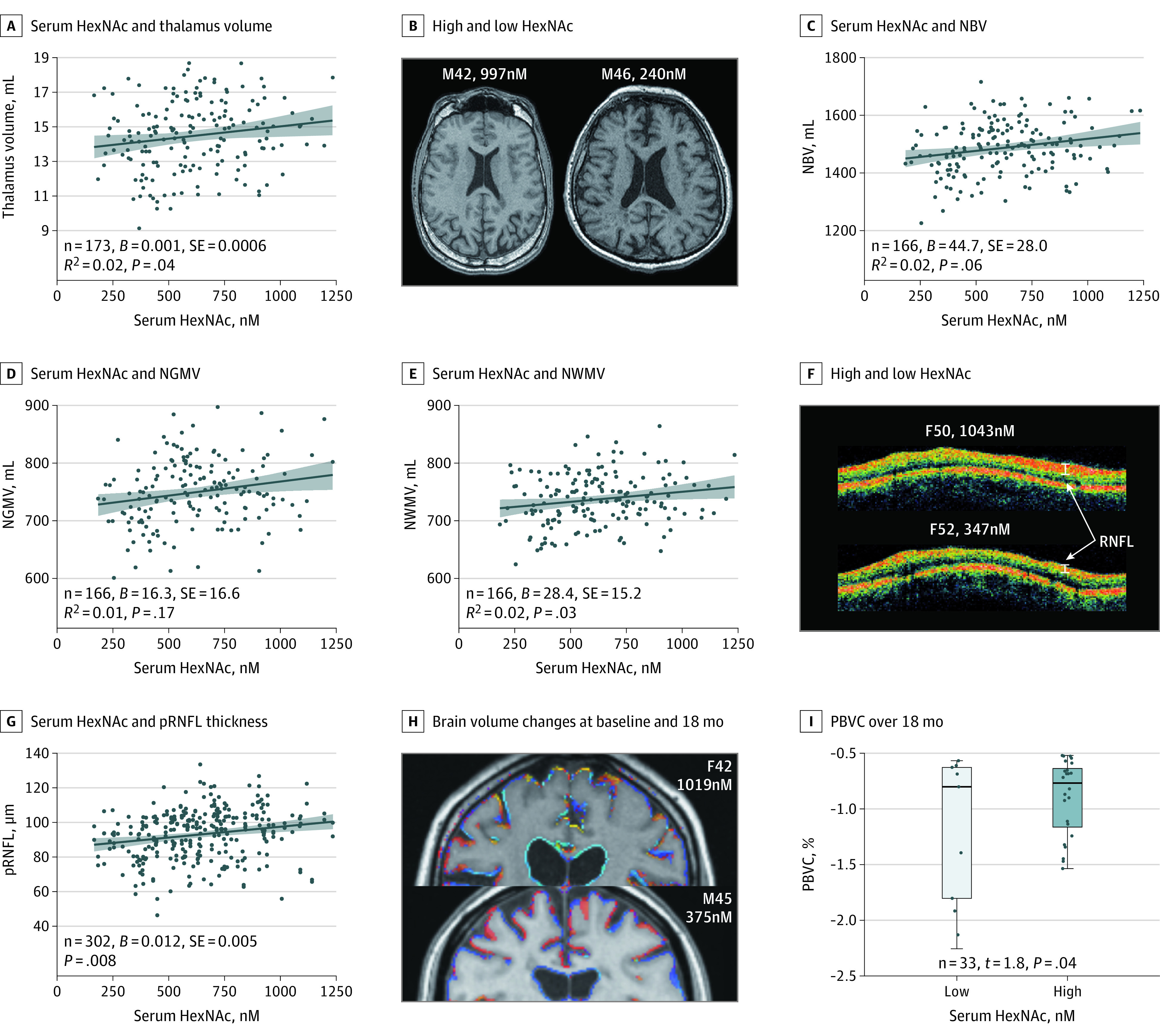
Correlation of Serum *N*-Acetylhexosamine (HexNAc) Level With Imaging Markers of Neurodegeneration A, Correlation of serum HexNAc level with thalamus volume by magnetic resonance imaging (MRI) of the confirmatory cohort. B, Example MRI images of patients with high and low HexNAc levels, labeled by sex (male = M), age in years, and HexNAc concentration. C, Correlation between serum HexNAc concentration and normalized whole-brain volume (NBV). D, Correlation between serum HexNAc concentration and normalized gray matter volume (NGMV). E, Correlation between serum HexNAc concentration and normalized white matter volume (NMWV). F, Assessment of retinal nerve fiber layer (RNFL) thickness by peripapillary ring scan optical coherence tomography (OCT) sample images of patients with high (top) and low (bottom) HexNAc concentration (both lacked history of optic neuritis). G, Correlation of HexNAc concentration with peripapillary RNFL (pRNFL) thickness by OCT in the confirmatory cohort. H, Example MRI outputs comparing brain volume changes at baseline (0 months) vs 18 months by SIENA in 1 patient with high (top) and 1 patient with low (bottom) HexNAc concentration at baseline. Blue indicates regional brain volume increase, whereas yellow indicates moderate and red strong regional brain volume loss. I, The percentage change in brain volume (PBVC) over 18 months by MRI between patients with low (gray) and high (light blue) HexNAc concentration at baseline using a median divider is compared using linear models, including baseline NBV, age, and sex as covariates. Coefficient *B*, SE, and *R*^2^ are from linear regression models correcting for age and sex (A-C, E) or generalized estimating equation models correcting for age and sex (G). The Spearman rank correlation ρ was used for analysis (C). The dark blue lines in regression models represent coefficients from noncorrected models, and the light blue areas show the 95% CI. The serum HexNAc levels are measured in units of nanomolar.

To evaluate whether HexNAc is associated with neuroaxonal damage in MS, we used MRI scans of the brain and OCT scans of the retina in the confirmatory cohort. Atrophy of the thalamus is an early and sensitive measure of neurodegeneration in MS,^40^ and lower serum HexNAc concentrations were associated with reduced thalamic volume, with age and sex as covariates (*P* = .04; Figure 4A and B). Likewise, reduced white matter volume (normalized white matter volume) was associated with lower serum HexNAc concentrations (*P* = .03), while whole-brain volume (normalized brain volume; *P* = .06) and gray matter volume (normalized gray matter volume; *P* = .163) were not significantly associated with lower serum HexNAc concentrations (Figure 4C-E). In contrast to brain volume, RNFL thickness provides a more stable measure of neuroaxonal damage in MS over a broad age range because it remains relatively constant in healthy adults until the age of approximately 50 years.^41^ Lower HexNAc serum levels were associated with more severe retinal axonal degeneration as measured by peripapillary RNFL (*B* = 0.012, *P* = .008; Figure 4F and G), with age and sex as covariates. Analysis by MS subtype revealed no association within the RRMS or PMS subtypes; however, this finding may be limited by lack of power and/or covariance.

To examine whether HexNAc levels are associated with brain atrophy over time, patients displaying pathologic brain volume loss detected on repeated brain MRI scans at 18 months (ie, percentage change in brain volume, ≥0.52%)^37^ were split into 2 groups based on the median HexNAc level. Patients with low HexNAc levels at baseline showed a greater decrease in brain volume than those with high HexNAc levels at baseline, with age, sex, and baseline normalized brain volume as covariates (n = 33, *t* = 1.8, *P* = .04; Figure 4H and I). Together, these data are consistent and reveal the association of low serum HexNAc levels with both clinical disability and multiple measures of neurodegeneration in MS.

## Discussion

Here we report that GlcNAc plus its stereoisomers are markedly reduced in PMS in 2 independent cohorts and that serum HexNAc levels are correlated with clinical disability and neuroimaging markers of neurodegeneration. Understanding immune mechanisms in RRMS has led to the development of multiple drugs that are successfully used clinically.^42^ In contrast, immunomodulatory drugs shown to be effective in RRMS are rarely effective in PMS.^43^ There is a significant deficiency in our knowledge of the mechanisms associated with PMS or, more broadly, of relapse-independent disease progression, which is observable throughout the disease course.^3,44^ Mechanisms associated with progressive disease in MS may include direct immune-mediated damage, failure of remyelination, and/or immune-independent neuroaxonal toxic effects.^45^ Ocrelizumab and siponimod, drugs recently approved for PPMS^46^ and active SPMS,^47^ mainly target residual neuroinflammation, whereas chronic demyelination and progressive neurodegeneration lack effective therapies.^48^ However, there are few data on biomarkers that allow detection of PMS, and diagnosis depends largely on clinical observation and, more recently, on potential imaging biomarker candidates.^49,50^ In contrast, molecular biomarkers are scarce and comprise complex type 2 biomarkers, which are difficult to interpret regarding their pathogenic relevance.^51,52^ Identifying simple molecular biomarkers for PMS that may facilitate early diagnosis, assess treatment effectiveness, and/or promote clinical development of novel treatment approaches is an unmet clinical need.^53^ Our data suggest that serum HexNAc levels should be further investigated as a potential biomarker for assessing MS patients at risk of disease progression. The clinical utility of biomarkers is often based on the AUC from ROC curves. The AUC for HexNAc (0.74-0.91) compares favorably with other well-established biomarkers, such as blood pressure, smoking, and cholesterol level for cardiovascular disease risk (AUC, 0.72-0.74).^54^ However, additional prospective clinical studies are required to substantiate the clinical utility as a biomarker and further clarify the potential physiological factors, such as diet, ethnicity, fasting status, and physical activity.

Mechanisms associated with neurodegeneration in MS may include direct immune-mediated damage, failure of remyelination, and/or immune-independent neuroaxonal toxic effect.^45^ *N*-glycan branching is a primary molecular mechanism that regulates cell surface protein activity in many diverse cells, thereby being involved in pleiotropic effects in multiple cell types relevant to MS. In both mice and human ex vivo studies, GlcNAc and/or *N*-glycan branching display significant immunomodulatory activity, suppressing mouse models of inflammatory demyelination by independently inhibiting both proinflammatory type 1 and type 17 helper T-cell responses^4,6,11,15,16,17,55^ as well as proinflammatory innate B-cell activity.^13^ *N*-glycan branching also acts as the ligand for galectins,^56^ which have been implicated in regulating microglial activity in animal models of MS.^57^ Human genetic sequence variants associated with *N*-glycan branching in T cells, including interleukin 7 receptor α, interleukin 2 receptor α, *MGAT1*, *MGAT5*, and vitamin D_3_, have been identified as MS risk factors.^20,21,22,23^ A recent study in mouse models observed that *N*-glycan branching directly drives new myelin formation from oligodendrocyte precursor cells and that oral GlcNAc prevents neuroaxonal damage to demyelinated axons by crossing the blood-brain barrier and promoting myelin repair.^19^ In humans, severe central nervous system hypomyelination results from loss-of-function variants in *PGM3*, a gene required to generate branched *N*-glycans from GlcNAc.^27^ *N*-glycan branching deficiency in PL/J mice promotes a spontaneous clinical syndrome that mimics PMS, characterized by inflammatory demyelination as well as axonal damage and neuronal death.^17^ Finally, neuronal survival in mice is directly regulated by *N*-glycan branching, as neuron-specific deficiency of the Mgat1 *N*-glycan branching enzyme results in spontaneous neuronal apoptosis in vivo and neurologic deficits.^24^ Thus, GlcNAc deficiency has the potential to be autonomously associated with multiple cellular mechanisms in diverse cell types critical to MS pathologic characteristics and neurodegeneration (namely, T cells, B cells, microglia, oligodendrocytes, and neurons). Although this broad mode of action is counterintuitive for a disease-specific mechanism, it is intriguing to postulate that GlcNAc deficiency may be associated with disease progression in MS through a combination of these mechanisms. However, additional clinical studies are necessary to pursue this hypothesis and better define the potential role of GlcNAc and *N*-glycan branching in clinical MS.

It is unclear why patients with PMS may have HexNAc deficiency. Sources of endogenous serum HexNAc include turnover of glycoconjugates as well as food, with the latter comprising glycan chains that may be hydrolyzed and/or catabolized in the intestine by microbiota. Interindividual variance in diet and gut microbiota may be significantly associated with HexNAc serum levels.^58^ Tissue damage may also be associated with serum levels. For example, GlcNAc is associated with the architecture of extracellular matrices, particularly hyaluronan, which accumulates in demyelinated lesions of patients with MS.^59^ Hyaluronan chains are large glycan polymers with a molecular weight of approximately 1.0 × 10^6^ Da in chronic demyelinated lesions. Because GlcNAc represents approximately half the mass of hyaluronan, chronic excessive hyaluronan synthesis in demyelinated lesions may lower the availability of GlcNAc locally and in serum.

### Strengths and Limitations

Our study has several strengths. Molecular biomarker studies in MS have led to several high-profile failures, in which the initial findings could not be independently verified, often with biomarkers of unclear mechanistic relevance to MS. In contrast, GlcNAc and *N*-glycan branching have been intensely studied as already summarized, and multiple cellular pathways important to progression and neurodegeneration in MS have been identified with different levels of evidence. Moreover, we used multiple methods to minimize potential bias as a source of error in our investigations. The LC-MS/MS analysis of serum HexNAc was performed in a blinded fashion by an independent investigator who was not involved in the study design and objective. We confirmed our main finding in a second cohort, which was managed in a different MS center in a different country by a different team of clinician-scientists. Third, all of the investigators of diagnostic, clinical disability, and neuroimaging measures in the confirmatory cohort were blinded to serum HexNAc levels.

This study also has some limitations. One potential source of bias is that both cohorts were derived from academic tertiary referral centers, and thus the data may not reflect a real-world distribution. In line with this, our cohorts lack racial/ethnic diversity. We therefore abstain from making further sensitivity or specificity analyses investigating HexNAc as a potential biomarker, which needs to be addressed in future studies. An additional limitation of the study is identifying HexNAc as opposed to GlcNAc in serum. Because the targeted LC-MS/MS method used in this study does not separate stereoisomers of *N*-acetylhexosamines (GlcNAc, GalNAc, and ManNAc), alterations of 1 or more of the stereoisomers may be associated with the observations reported herein. However, unlike GlcNAc, the GalNAc and ManNAc isomers lack mouse or human data implicating a role in MS. Additional approaches to distinguish GlcNAc from its stereoisomers within serum are required to clarify these issues and better define the specific role for GlcNAc in MS. As another limitation, associations in the main analyses comparing HexNAc serum concentrations between patients with RRMS and those with PMS were large, but subgroup analyses (eg, comparing patients with PPMS and those with SPMS) may have been underpowered. This is equally the case in the correlation analyses with clinical and imaging parameters of disease, for which strong covariance and clinical and imaging markers typically demand larger sample sizes. Furthermore, we did not investigate HexNAc in association with advanced MRI parameters (myelination and microstructural damage) or macular OCT parameters, which were not available because of the older MRI and OCT technologies used. The mostly cross-sectional design also prevents definitive conclusions about the predictive power of serum HexNAc for MS disease development.

## Conclusions

Our study identifies reduced serum HexNAc levels as a potential molecular biomarker of PMS. These new clinical data build on preclinical, human ex vivo, and genetic research establishing a role of *N*-glycan branching and its metabolite GlcNAc in MS-relevant disease processes, including inflammation but, importantly, also myelination and neurodegeneration. It also suggests that a fundamental molecular process, *N*-glycan branching, may be altered in PMS, separating it from RRMS. Further studies are required to develop our understanding of the relevance of *N*-glycan branching and GlcNAc in PMS and of the clinical research and/or clinical management of disease progression in MS.

## Notes

Supplement. eMethods. eReferences. eTable 1. Discovery Cohort eTable 2. Confirmatory Cohort
