# Supplementary material for: Association of a Marker of N-Acetylglucosamine With Progressive Multiple Sclerosis and Neurodegeneration
Source: JAMA Neurol. 2021 May 10;78(7):1–11. doi: 10.1001/jamaneurol.2021.1116 (PMC8111565; doi:10.1001/jamaneurol.2021.1116)
Supplement: Supplement. — eMethods. eReferences. eTable 1. Discovery Cohort eTable 2. Confirmatory Cohort [file jamaneurol-e211116-s001.pdf]

Supplementary Online Content

Brandt AU, Sy M, Bellmann-Strobl J, et al. Association of a marker of *N*-acetylglucosamine with progressive multiple sclerosis and neurodegeneration. *JAMA Neurol.* Published online May 10, 2021. doi:10.1001/jamaneurol.2021.1116

eMethods.
eReferences.
eTable 1. Discovery Cohort
eTable 2. Confirmatory Cohort

This supplementary material has been provided by the authors to give readers additional information about their work.

© 2021 Brandt AU et al. *JAMA Neurology*.

eMethods

Patient consent

The local ethics committees in Irvine and Berlin approved the studies for the initial and confirmatory cohorts, respectively. Patients in the confirmatory study consented for use of their data outside the respective trials, in which their data was acquired. Studies were conducted in conformity with the 1964 Declaration of Helsinki in its currently applicable version, following Good Clinical Practice guidelines. All study participants gave written informed consent.

Clinical Scoring

Clinical examination was performed according to Kurtzke's Expanded Disability Status Scale (EDSS).[1] Additionally, disability was assessed using the MS Functional Composite (MSFC), comprising the timed 25-ft walk test (T25FW), the 9-hole peg test (9-HPT) and the 3-second Paced Auditory Serial Additions Test (PASAT).[2] MSFC z-scores were calculated according to the MSFC Administration and Scoring Manual. MS Severity Scores (MSSS) were calculated from disease duration and EDSS.[3]

Magnetic Resonance Imaging (MRI)

MRI was performed at 1.5 Tesla using three-dimensional T1-weighted magnetization prepared rapid acquisition and multiple gradient echo sequences (MPRAGE; T1w) and axial T2-weighted (T2w) sequences. Images were either acquired on a Sonata MRI (Siemens Medical Systems, Erlangen, Germany) with TE 4.38ms, TR 2,110ms, TI 1.1ms, flip angle 15° and isotropic resolutions 1 mm^3 for T1w, and multiecho TSE with TE 81ms, TR 5,780ms, 150° flip angle, resolution $0.5x0.5x3mm^3$, no gap for T2w, or on an Avanto MRI (Siemens Medical Systems, Erlangen, Germany) with TE 3.09ms, TR 1,900ms, TI 1.1ms, flip angle 15° and isotropic resolutions $1mm^3$ for T1w, and 3D TSE with TE 175ms, TR 3,000ms, flip angle 120°, isotropic resolutions $1mm^3$ for T2w.

Thalamic volume was determined as summary volume from both hemispheres using FIRST (FSL 5.0, FMRIB Software Library, Oxford, UK)[4] on MPRAGE scans and normalized using a brain-size normalization factor output from FSL SIENAX (FSL, FMRIB Software Library, Oxford, UK)[5] for each individual brain. Brain volumes were determined using MPRAGE scans with the FSL 5.0 pipeline SIENAX. For pre-processing, MRI were cropped to a robust field of view and registered to MNI standard space implemented in FSL with subsequent correction for bias field

© 2021 Brandt AU et al. *JAMA Neurology.*

non-uniformity using the N3-algorithm in the MIPAV package version 5.4.2. SIENAX computes global brain volume (NBV) as well as grey matter (NGMV) and white matter (NWMV) volume estimates normalized with respect to the individual's head size, accounting for inter-individual variability. Two-time point percentage brain volume change (PBVC) was estimated with SIENA, part of FSL,[5] in those patients for which 18-month follow-up data was available. Patients with pathological PBVC were determined by established cutoff of 0.52% annual loss (95% specificity).[6]

Optical Coherence Tomography (OCT)

Retinal nerve fiber layer thickness (RNFL) from both eyes was measured with a Stratus OCT, software version 4.0 (Carl Zeiss Meditec, Dublin, CA, USA) using the fast RNFL 3.4 protocol as previously described.[7] Patients were examined without pupil dilation. Only images with acceptable quality were included in the analysis, defined as visually even signal distribution, a reflectance signal strong enough to identify the RNFL layer borders, correct centration, and signal strength of ≥7 of 10. Images with erroneous RNFL segmentation were excluded from analysis.

Targeted Liquid chromatography - tandem mass spectroscopy (LC-MS/MS)

All serum samples were analyzed in a blinded fashion by the central mass spec laboratory in Toronto. The operator performing the measurements (JP) was not involved in study design or informed about the scientific objectives. Results were then sent to the main investigators, who performed statistical analysis. Serum samples for metabolomics analysis were prepared as described previously.[8] Briefly, 50 µL serum (stored at -80°C) and 200µl ice cold extraction solvent (40% acetonitrile: 40% methanol: 20% H_2O), were vortexed for 2 minutes, then shaken in an Eppendorf shaker (Thermomixer R) at 1400 rpm, 4°C for 1 hour and centrifuged at 4°C for 10 minutes at ~18,000 x g in an Eppendorf microfuge. Supernatants were transferred to a clean tube and evaporated in a Speedvac (Acid-Resistant CentriVap Vacuum Concentrators, Labconco). Dried samples were stored at -80°C. Samples were resuspended in 100 µl of water containing the Internal Standards D^{7}-Glucose at 0.2 mg/mL and H-Tyrosine at 0.02 mg/ml. Samples were resolved by LC-MS/MS, in negative mode at the optimum polarity in MRM mode on an electrospray ionization (ESI) triple-quadrupole mass spectrometer (AB Sciex 4000Qtrap, Toronto, ON, Canada). MultiQuant software (AB Sciex, Version 2.1) was used for peak analysis and manual peak confirmation. The results, expressed as area ratio (area of analyte/area of internal standard), were exported to Excel, and analyzed with MetaboAnalyst 3.0.[30] Standard curves were prepared by adding increasing concentrations of GlcNAc or N-Acetyl-D-

© 2021 Brandt AU et al. *JAMA Neurology*.

[UL-$^{13}C_6$]glucosamine ([UL$^{13}C_6$] GlcNAc) (Omicron Biochemicals, Indiana) to 50 µl aliquot of control serum (**Fig. 1B**). This way we were able to create a calibration curve for HexNAc serum levels, obtaining absolute values rather than relative concentrations (**Fig. 1C**). Analysts were blinded in regard to sample origin (control or patient).

Statistical Analysis

Statistical analyses were performed with R Project version 3.5.3. Sample sizes were based on convenience samples. Correlation between age or sex and serum HexNAc levels were analyzed with linear regression models for age and Welch's t-test for sex in HC. Group differences between HC, RRMS and PMS patients were analyzed using linear models with age as covariate. To combine results of group comparisons in the discovery and confirmatory cohorts, we used Fisher's combined probability test, which combines p-values using their logarithmic transformation. Association between treatment status and serum HexNAc was assessed by the Kruskal-Wallis test. For boxplots, the solid middle line represents the median. The lower and upper hinges correspond to the 25th and 75th percentiles. The upper and lower whisker extends from the hinge to the distant value no further than 1.5*IQR from the hinge (where IQR is the inter-quartile range, or distance between 25th and 75th percentiles). Receiver Operating Characteristics curves (ROC) were used to quantify serum HexNAc's ability to discriminate between RRMS and PMS. Correlations between serum HexNAc level and clinical scores or imaging parameters were analyzed using linear regression models with HexNAc serum level as an independent parameter, except for EDSS and lesion measurements, which were analyzed using non-parametric Spearman's Rho analyses (EDSS because of the measure's ordinality, and lesion measurements because of their non-normal or skewed distributions i.e. as count variables). RNFL correlations were analyzed with generalized estimating equation models (GEE) accounting for inter-eye within-patient effects and using serum HexNAc level as an independent parameter. All correlation analyses based on parametric models were corrected for age and sex. Partial R^2 was calculated to only include serum HexNAc-attributable variance in these models. PBVC between month 18 and baseline was compared using linear models with baseline NBV, age and sex as covariate. Serum HexNAc concentration before and during oral treatment with GlcNAc were compared using one-sided paired Wilcoxon signed-rank tests, comparing the mean of the weeks before treatment to the mean of the weeks under treatment. Data from discovery and confirmatory cohorts were examined for non-normal distributions by visual inspection of histograms and calculation of skewness and

© 2021 Brandt AU et al. *JAMA Neurology*.

kurtosis. In case data was missing, data was not amended (sample size is indicated in each figure). Significance in all tests was established at $p<0.05$, whereas a trend was considered in $p<0.1$. After initial two-sided testing of HexNAc serum concentration in HC and in RRMS vs. PMS patients, all further tests were one-sided testing an association of serum HexNAc levels with worse disease outcomes.

© 2021 Brandt AU et al. *JAMA Neurology*.

eReferences

1 Kurtzke, J. F. Rating neurologic impairment in multiple sclerosis: an expanded disability status scale (EDSS). *Neurology* **33**, 1444-1452 (1983).

2 Cutter, G. R. *et al.* Development of a multiple sclerosis functional composite as a clinical trial outcome measure. *Brain* **122 (Pt 5)**, 871-882, doi:10.1093/brain/122.5.871 (1999).

3 Roxburgh, R. H. *et al.* Multiple Sclerosis Severity Score: using disability and disease duration to rate disease severity. *Neurology* **64**, 1144-1151, doi:10.1212/01.WNL.0000156155.19270.F8 (2005).

4 Patenaude, B., Smith, S. M., Kennedy, D. N. & Jenkinson, M. A Bayesian model of shape and appearance for subcortical brain segmentation. *Neuroimage* **56**, 907-922, doi:10.1016/j.neuroimage.2011.02.046 (2011).

5 Smith, S. M. *et al.* Accurate, robust, and automated longitudinal and cross-sectional brain change analysis. *Neuroimage* **17**, 479-489 (2002).

6 De Stefano, N. *et al.* Establishing pathological cut-offs of brain atrophy rates in multiple sclerosis. *J Neurol Neurosurg Psychiatry* **87**, 93-99, doi:10.1136/jnnp-2014-309903 (2016).

7 Dorr, J. *et al.* Association of retinal and macular damage with brain atrophy in multiple sclerosis. *PLoS One* **6**, e18132, doi:10.1371/journal.pone.0018132 (2011).

8 Abdel Rahman, A. M., Ryczko, M., Pawling, J. & Dennis, J. W. Probing the hexosamine biosynthetic pathway in human tumor cells by multitargeted tandem mass spectrometry. *ACS Chem Biol* **8**, 2053-2062, doi:10.1021/cb4004173 (2013).

© 2021 Brandt AU et al. *JAMA Neurology*.

eTable 1. Discovery Cohort

		HC	RRMS	PMS
**Subjects**	*n*	66	33	21
PPMS	*n*			*2*
SPMS	*n*			*19*
**Age (years)**	Mean (SD)	42 (20)	50 (11)	55 (7)
**Sex (M/F)**	*n/n*	28/38	8/25	7/14
**Treatment**	*Glatiramer Acetate*		20	7
	Interferons		10	6
	Corticosteroids		3	7
	None		0	1

© 2021 Brandt AU et al. *JAMA Neurology*.

eTable 2. Confirmatory Cohort

		RRMS	PMS
**Subjects**	*n*	125	55
PPMS	*n*		*23*
SPMS	*n*		*32*
**Age (years)**	Mean (SD)	40 (9)	49 (8)
**Sex (M/F)**	*n*/*n*	42/83	31/22
**Time since diagnosis (months)**	Mean (SD)	113 (81)	129 (101)
**EDSS**	Median (Min - Max)	2 (0-6)	6 (3-8)
**MSSS**	Mean (SD)	3.2 (2.3)	6.8 (1.7)
**T25FW (sec)**	Mean (SD)	5.02 (1.82)	11.24 (7.61)
**9-HPT dominant hand (sec)**	Mean (SD)	20.65 (5.39)	31.22 (16.81)
**9-HPT non-dominant hand (sec)**	Mean (SD)	20.99 (4.51)	32.50 (13.66)
**PASAT-3 (from 60)**	Mean (SD)	48.06 (11.98)	41.67 (11.92)
**Thalamus Volume (ml)**	Mean (SD)	13.72 (2.13)	14.83 (1.88)
**NBV (1,000 x ml)**	Mean (SD)	1,510,627 (84,548)	1,429,843 (73,349)
**NGMV (1,000 x ml)**	Mean (SD)	765,433 (53,471)	711,502 (44,458)
**NWMV (1,000 x ml)**	Mean (SD)	745,193 (43,165)	718,341 (41,592)
**RNFL (µm)**	Mean (SD)	94.6 (13.4)	87.0 (11.4)

© 2021 Brandt AU et al. *JAMA Neurology*.
